# Evaluation of the Effects of Whey Protein and *Moringa* *Oleifera* Leaves Extract Mixture on Osseointegration in Rabbits

**DOI:** 10.61186/ibj.4025

**Published:** 2023-12-17

**Authors:** Nawar Bahjet Kamil, Nada M.H. AL-Ghaban

**Affiliations:** Department of Oral Diagnosis, College of Dentistry, University of Baghdad, Baghdad, Iraq

**Keywords:** Dental implants, Insulin-like growth factor I, Moringa oleifera, Osseointegration, Whey proteins

## Abstract

**Background:**

Osteogenic, antioxidant and anti-inflammatory effects of Whey protein and *M. oleifera* gel prompted us to evaluate their role alone or in combination on osseointegration in rabbits.

**Methods:**

In this study, 24 titanium implants were inserted in the femurs of six rabbits. One implant was placed without treatment, and another one was coated with a mixture of whey protein and *M. oleifera* gel for each side. The animals were divided into two groups of 2- and 6-week intervals and evaluated using histopathological and immunohistochemical techniques.

**Results:**

Histological evaluation revealed a significant difference between the experimental and the control groups after two weeks in osteoblast and osteocyte counts. The experimental group had mature bone development after six weeks of implantation, while the control group had a woven bone. Immunohistochemical results showed that the experimental group, compared to the control group, exhibited early positive expression of osteoblast cells at two weeks after the experiment. Based on histopathological observations, the experimental group showed a tiny area of collagenous fiber in 6^th^ week after the implantation.

**Conclusion:**

A mixture of whey protein and *M. oleifera *could accelerate osseointegration and healing processes.

## INTRODUCTION

Osseointegration, the direct contact between implant and bone surfaces, frequently occurs in two dimensions. The proportion of bone-to-implant contact and the pressure required to dislodge the implant from the bone act as a biological readout for osseointegration^[^^1^^]^. For dental implants, which predominantly consist of titanium, osseointegration is crucial. Surface etching and sandblasting of titanium-based dental implants can induce rough surfaces, which appear to be ideal for osseointegration^[^^2^^]^. 

Cow’s milk is a rich source of a premium protein known as whey protein. Casein and whey proteins comprise around 80% and 20% of the protein components of the milk, respectively^[^^3^^]^. Whey has a protein content of less than 1% that mostly consists of lactoglobulin and lactalbumin. It also contains small amounts of glycoproteins such as lanolin, lactoferrin, transferrin, and lactoperoxidase, as well as comprises proteases, peptones, immunoglobulins, and bovine serum albumin^[^^4^^]^. Whey protein inhibits the disruptive effects of stress, as a part of its antioxidant action related to glutathione synthesis. Bioactive peptides are released from the whey proteins, leading to an increased intracellular glutathione level and decreased production of IL-8, a cytokine involved in respiratory diseases^[^^5^^]^. Considering that the whey protein includes various components with the ability to prevent the destructive activity of toxins, germs, and viruses, it can stimulate immune cells and/or prevent infection. Lactoferrin and its peptide derivatives, lactoperoxidase, lactoferricin, and sphingolipids, are a number of the components with antibacterial activity^[^^6^^]^. Lactoferrin, lanolin, and lactoglobulin have also shown an inhibitory effect against HIV type 1^[^^7^^]^. β-lactoglobulin is an effective agent in preventing the spread of HIV and genital herpes virus infections. It can inhibit parasitic protozoa, fungi, yeast, Gram-positive and Gram-negative bacteria^[^^8^^]^. 

The leaves, seeds, bark, roots, and flowers of the *M.* *oleifera* plant are widely used in traditional medicine, owing to their low side effects, safety to use, and strong antioxidant activity. Yet no adverse outcomes have been reported for this plant in human studies^[^^9^^]^. The leaves of *M.* *oleifera* consist of vitamins (A, B6, and C), iron, riboflavin, proteins, and magnesium; however, the antioxidant activity of the extract of this part of the plant has rarely been investigated. The *M.* *oleifera* also contains a flavonoid that has lately been advocated for the prevention and treatment of COVID-19^[^^10^^]^. Flavonoids and bioactive substances found in *M.* *oleifera*, help protect people from chronic degenerative diseases. The main flavonoids of the plant are quercetin and kaempferol derivatives, which have anti-inflammatory properties in the form of glycosides, malonyl glycosides, acetyl glycosides, and succinyl glycosides^[^^11^^]^. 

Growth factors have active participation in the formation and regeneration of bones. These chemicals control osteoprogenitor cell migration, their proliferation and differentiation to mature osteoblasts, type I collagen production, and matrix apposition through autocrine and paracrine signaling processes^[^^12^^]^. IGF-I is important in the regulation of new bone formation. It is a small peptide with a similar structure to insulin that acts as a regulator of skeletal growth. IGF-I is synthesized in the liver under the effect of growth hormone and is bound to IGF-binding protein-3. Circulating IGF-I affects longitudinal bone growth. Osteoblasts can locally synthesize IGF-I in response to PTH, which mediates its anabolic effects on bone. IGF-I and -II are stored in the extracellular matrix of bone and serve as the most abundant growth factors^[^^13^^]^. 

The present study was conducted to histologically, histochemically and immunohistochemically evaluate the effect of whey protein and *M. oleifera* gel combination on osseointegration. 

## MATERIALS AND METHODS

**Study samples**

Six adult healthy male white New Zealand rabbits, aged between 9 and 13 months and weighing 2.5-3 kg, were used in the study. A total of four dental implants, two in the right femur and two in the left femur, were placed in each rabbit by surgical operation. One of the implants placed on each side was left untreated, and the other was covered with a mixture of 0.25 mg of whey protein and 0.25 ml of *M.* *oleifera* gel^[^^14^^]^. The gel was prepared by drying, hardening, and grinding the *M.* *oleifera* leaves^[^^15^^]^.

**Surgical process and slide preparation**

Intermittent drilling and continuous irrigation were applied by physiological saline to prevent heating of the surgical area. Then 1.8-mm diameter holes were created in the place of the implants, with the help of a micro motor fitted with a round bur. A 20-mm gap was left between the two holes. Tissue debris in the operation regions was removed by irrigating the area using physiological saline^[^^16^^]^. 

**Slide preparation**

At the end of the 2^nd^ and 6^th^ weeks after implantation, three animals were sacrificed in each period. Bone tissue samples containing the implant site were processed through routine histotechniques, by immersing in paraffin blocks and then sectioning. The sections were stained by H&E and Masson’s trichrome. The specimens were finally examined under a light microscope, and the regions surrounding the implants were evaluated histologically and histo-morphometrically. Bone marrow and trabecular area were expressed in µm^2^, while the newly formed bone area was expressed as a percentage of the measured tissue area (1.45 mm^2^) according to the following equation: newly formed bone area = measured bone area/measured tissue area (×100)^[^^17^^]^, using Image J software. Osteoblast and osteocyte counts were determined by considering the mean numbers in the bone tissue with dimensions of 1.41 × 1.05 mm located in the first three threads of the implant, under ×40 magnification^[^^18^^,^^19^^]^. For the immunohistochemical demonstration of IGF-I, a polyclonal primary antibody (Abcam, UK) was employed. Each sample was evaluated for the intensity of the brown cytoplasmic DAB reaction product. The intensity of stained cells scored from 0 to 3 as follows: (0) negative, (1) weak, (2) moderate, and (3) strong. The positive expression stained cells scored from 0-4, score (0) belonged to the frequency of positive cells less than 2%, score (1) was between 2 and 10%, score (2) between 11 and 25%, score (3) between 26 and 50%, and score (4) was greater than 50%, respectively. Total immunoreactivity was determined as the sum of the staining intensity and the percentage of immune-positive cells^[^^20^^]^. In Masson’s trichrome-stained specimens, osteoid tissue was examined because the tissue was rich in type I collagenous fibers. 

**Fig. 1:**
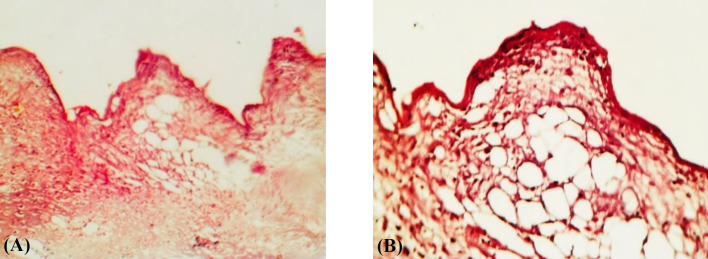
Sections of the defect sites in the study groups at the end of 2^nd^ week after implantation. (A) In the control group, areas between the threads of the implants are occupied mainly with loose connective tissue rich in capillary blood vessels and adipocytes. (B) In the experimental group, a narrow fibrocartilage tissue, with a small number of osteoblastic cells, in the areas adjacent to the implant threads was observed. The sections were observed under a light microscope with magnitudes ×200 and ×400

**Statistical analysis**

 The data were analyzed using the SPSS 25 software. The mean, median, standard deviations and *p* values of each parameter were determined. *P* values under or equal to 0.05 were considered statistically significant.

## RESULTS

**Histochemical findings**

**Fig. 2:**
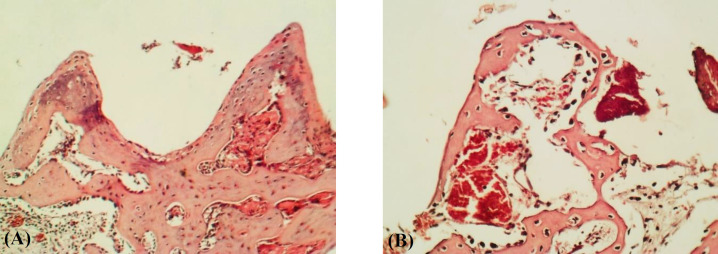
Sections of the defect sites in the study groups at the end of the 6^th^ week after implantation. The areas between the threads of the implant were filled with trabecular bone. (A) In the experimental group, bone is more organized, and trabeculae are greater than (B) the control group. The sections were observed under a light microscope with magnitudes ×200 and ×400

 At the end of the 2^nd^ week following implantation, histological findings of the implant areas in the control group showed that the area between the implant threads was substantially filled with loose connective tissue rich in capillary blood vessels and fat cells. In addition, no broad fibrocartilage tissue was observed in the area (Fig. 1A). In contrast, in the experimental group, loose connective tissue such as fibrocartilage tissue consisting of osteoblastic and osteocytic cells, have mainly filled the spaces between the implant threads at the end of the 2^nd^ week (Fig. 1B). At the end of the 6^th^ week after implantation, the regions between implant threads were mostly filled with osseous tissue in both study groups (Fig. 2). However, the experimental animals (Fig. 2A) exhibited an advanced production of mature, trabecular bone relative to the control animals (Fig. 2B). In the same period, the experimental group had higher osteocyte cell number, newly formed bone rate, and trabecular area as compared to the control group. However, osteoblastic cell numbers and bone marrow area were lower than those of the control. Moreover, the experimental group had significantly (*p* < 0.01) higher values in all parameters, except for the bone marrow area, at the end of the 2nd week and Osteoblastes number at the 6th week (Table 1). 

**Table 1:** Histomorphometric results of the study groups at the end of the 2^nd^ and 6^th^ week of the experiment

**Parameters**	**Groups**				
	**2** ^th ^ **week**			**6** ^th^ ** week**	
	**Control** **(X ** **± ** **SE)**	**Experimental** **(X ** **± ** **SE)**		**Control** **(X ** **± ** **SE)**	**Experimental** **(X ** **± ** **SE)**
Osteoblastic cell number (1.41 × 1.05 mm^2^ tissue area)	21.17 **± **1.02	46.02 **± **1.50^*^		34.25 **± **1.56^*^	20.20 **± **0.61
Osteocyte number (1.41× 1.05 mm^2^ tissue area)	8.98 **± **0.83	16.80 **±** 0.98^*^		22.98 **± **1.05	43.43 **± **1.34^*^
Newly formed bone rate (%)	2.02 **± **0.11	3.24 **± **0.13^*^		3.51 **± **0.17	5.29 **± **0.19^*^
Trabecular bone area (µm^2^)	0.35 **± **0.04	0.69 **± **0.06^*^		0.46 **± **0.04	0.90 **± **0.03^*^
Bone marrow area (µm^2^)	0.54 **± **0.04^*^	0.31 **± **0.02		0.41 **± **0.05^*^	0.21 **± **0.03

^ *^The differences between the mean values in the same line are statistically different (*p* < 0.01).

**IGF-I expression**

**Fig. 3:**
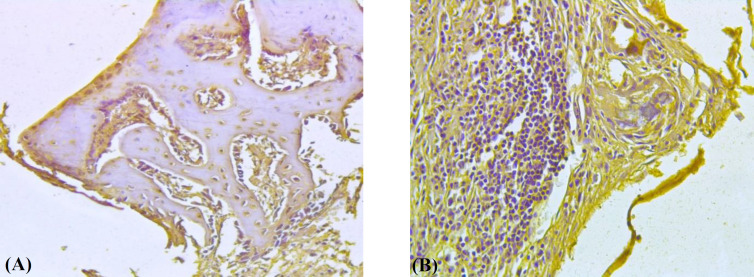
IGF-I immunohistochemical staining of the sections of the defect sites in the (A) experimental and (B) control groups at the end of 2^nd^ week of the experiment. (A) Positive reactions of the osteoblasts, osteoclasts, and osteocytes are definite in the experimental group. (B) Negative staining of the osteocytes and stromal cells is clear in the control group. The sections were observed under a light microscope with magnitudes ×200 and ×400

Results of the immunohistochemical investigations showed that osteoblast, osteoclast, osteocytes, and bone marrow cells of the control group did not have immune-positive reaction to IGF-I at both periods of the experiment (Fig. 3). In the experimental group, most of the investigated cells displayed IGF-I immune reactivity, except for bone marrow stromal cells (Fig. 3A). Similar results were also observed at the end of the 6^th^ week after implantation. Immune-positive cell scores of the experimental group were significantly (*p* < 0.05) higher than those of the control group at the end of the 2^nd^ and 6^th^ weeks of the experiment (Table 2).

**Findings of Masson’s trichrome-staining**

**Fig. 4:**
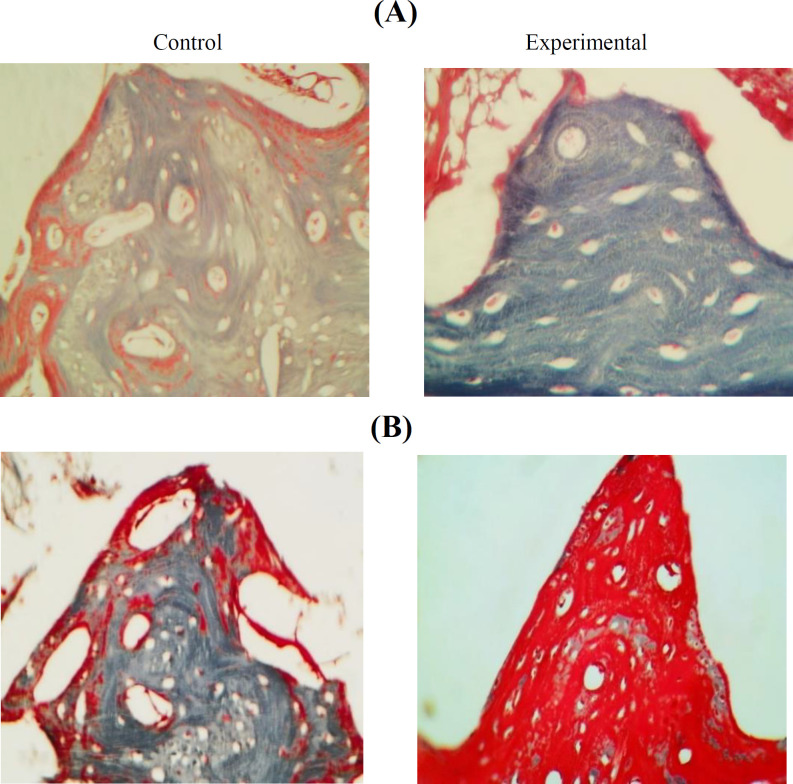
Sections of the defect sites in the study groups at the end of (A) 2^nd ^and (B) 6^th^ weeks. (A) Collagenous fiber intensities are seen in the fibrocartilage tissue. (B) Collagen staining intensity is higher in the control than in the experimental group. Masson’s trichrome stain. The sections were observed under a light microscope with magnitudes ×200 and ×400

 Results of histochemical findings showed that the two study groups had a sizable red-colored region that represented the amount of collagen in the second week (Fig. 4A). In the sixth week, the experimental group displayed a small region indicative of bone mineralization, whereas the control group exhibited a large area of collagen intensity (Fig. 4B). 

## DISCUSSION

Titanium and its alloys are the ideal bio-integrated materials for dental implants. The integration between the tissue and implants depends on bone quality and quantity, as well as implant material and loading conditions^[^^21^^]^. In the current study, a combination of *M. oleifera* and whey protein was used because the primary component of whey protein, namely lactoglobulin, can alter the immunity and acts as an antioxidant, anticancer, antiviral, and antibacterial agent^[^^22^^]^. Also, *M. oleifera* leaf extract is rich in tannins, saponins, alkaloids, and flavonoids, and it contains flavonoids that can promote osteoblast development and proliferation^[^^23^^]^. Interestingly, saponins influence osteogenic activity, thus promoting osteoblast proliferation, differentiation, and bone formation^[^^24^^]^.

At the end of the 2^nd^ week of the experiment, the histological analysis of the control group revealed that the regions between the screw threads were filled with loose connective tissue separated with a distinct line from the original bone. Furthermore, osteoclastic activity at the implant site was weak, and few bone spicules were observed in the implant area. This observation has also been demonstrated by Othman and Al-Ghaban^[^^25^^]^. Histological findings of the whey protein and *M. oleifera* gel-coated implant group showed that the newly formed bone tissue was surrounded by a narrow connective tissue containing woven bone islets, blood capillaries, collagenous fibers, and a small number of inflammatory cells. These findings are consistent with those of Al-Molla et al.^[^^16^^]^. 

**Table 2:** Results of the immunohistochemical investigations at the end of the 2^nd^ and 6^th^ weeks

**Cell types**	**Groups**				
	**Control ** **(X ** **± ** **SE)**			**Experiment ** **(X ** **± ** **SE)**	
	**End of the 2** ^nd^ ** week**	**End of the ** **6** ^th^ ** week**		**End of the 2** ^nd^ ** week**	**End of the 6** ^th^ ** week**
Osteocytes	3.50 ± 1.05	4.33 ± 1.03		5.17 ± 0.98^*^	3.5 ± 1.05
Osteoblastic cells	3.67 ± 0.82	4.17 ± 1.17		5.67 ± 0.52^*^	3.17 ± 1.17
Ossteoclastic cells	1.83 ± 0.75	2.17 ±0.41		2.67 ± 0.52^*^	2.0 ± 0.63
Bone marrow stromal cells	5.17 ± 0.75	3.83 ± 1.17		4.5 ± 1.05	2.5 ± 0.55

Based on our literature survey, the anti-inflammatory and antibacterial properties of whey protein and *M. oleifera* gel are attributed to their lactoferrin, lactoglobulin, and lactalbumin contents. These contents could produce a higher number of osteoblastic cells and osteocytes in the experimental group than in the control group at the end of the 2^nd^ week^[^^26^^]^. Sixth weeks after surgery, we observed the thickening and increasing number of bone trabeculae in the implant area in the experimental group. However, only a few newly formed bone trabeculae were found in the control group. These findings indicate that healing and bone formation accelerate in the experimental animals than in the control animals. In this regard, Davison et al. reported similar results^[^^26^^]^. Sixth weeks after implantation, in the 

control group, the implant threads were filled with woven bone, whereas in the experimental group, they were filled with mature bone, characterized by osteon formation. These observations can be attributed to the antioxidant properties of whey protein^[^^27^^]^ and *M. oleifera* gel^[^^28^^]^, as suggested by previous researchers^[^^29^^-^^33^^]^. Whey protein and *M. oleifera* extracts are thought to be superior sources for free cysteine, which boosts the production of glutathione and regulates bone cell differentiation while accelerating bone formation and decreasing bone marrow volume. At the end of the 6^th^ week after surgery in the experimental group, the bone marrow area significantly reduced compared to the control group, which supports the results obtained by Mohamed et al.^[^^34^^]^.

In the present study, IGF-I was detected by immunohistochemical method because osteocytes express significant amounts of IGF-I^[^^35^^]^, and disruption of the IGF-I gene in osteocytes impairs the development and growth of the bone. IGF-I is an important bone formation marker, related to PTH ^[^^36^^]^, and is a signaling mediator of PTH receptor in the regulation of osteocytes during bone formation^[^^37^^]^. IGF-I is expressed in the proliferating osteoblasts and osteoblast-like cells in the early phase of bone formation^[^^38^^]^. In our study, collagen synthesis, osteoblastic activity, and IGF-I scores of osteoblastic cells, osteocytes, and osteoclasts in the experimental group reached the highest level at the end of the 2^nd^ week, while all scores of the control group were low. At the end of the second postoperative week, the healing rate and bone formation in the experimental group were improved, and the osteocyte, osteoblast, and osteoclast levels at the implant site were found to be higher than those in the control group. These results are consistent with Duan et al.’s findings^[^^39^^]^. 

Type I collagenous fibers abundant in the osteoid tissue have been shown in Masson’s trichrome-stained sections^[^^40^^]^. At the end of the 2^nd^ week of our experiment, the observation of randomly oriented collagen fibers and a few osteocytes indicated the presence of osteoid tissue in the area and also the start of the formation of mature bone tissue. At this period, abundant collagen fibers were observed in the defect area filled with fibrocartilage tissue in the experimental group. At the end of day six, the defect area in the experimental group was filled with mature bone with little collagen; however, in the control group, it was largely filled with woven bone and osteoid tissue, which supports the findings of Shayegan et al.^[^^41^^]^.

## CONCLUSION

The pattern and progression of intramembranous osteogenesis can be observed by bone healing around the implant. In the implant area, fibrocartilage, osteoid tissue, woven bone, and lamellar bone are formed, respectively. As shown in this study, surface-treated implants with a mixture of whey protein and *M. oleifera* gel are well tolerated biologically. Due to earlier differentiation and proliferation of bone-forming cells, osseointegration is augmented by the new bone formation and mineralization that is accelerated and achieved on the surface treated implant sites. 

## DECLARATIONS

### Acknowledgments

The authors did not use artificial intelligence (AI)-assisted technologies in the production of submitted work.

### Ethical approval

This study was carried out by the animal experimentation ethical principles and approved by the Research Ethics Committee of Baghdad University Faculty of Dentistry, Baghdad, Iraq (ethical code: 531 in 30-6-2022).

### Consent to participate

Not applicable.

### Consent for publication

All authors reviewed the results and approved the final version of the manuscript.

### Authors’ contributions

NBK and NMA: contributed to the conception, design, analysis, and interpretation of data. 

### Data availability

All relevant data can be found within the manuscript. 

### Competing interests

The authors declare that they have no competing interests. 

### Funding

This research received no specific grant from any funding agency in the public, commercial, or not-for-profit sectors.

### Supplementary information

The online version does not contain supplementary material. 

## References

[1] Albrektsson T, Johansson C (2001). Osteoinduction, osteoconduction and osseointegration. Eur Spine J.

[2] Gittens RA, Olivares Navarrete R, Schwartz Z, Boyan, BD (2014). Implant osseointegration and the role of microroughness and nanostructures: lessons for spine implants. Acta Biomater.

[3] Wolfe RR (2000). Protein supplements and exercise. Am J Clin Nutr.

[4] Kilara A, Panyam D (2003). Peptides from milk proteins and their properties. Crit Rev Food Sci Nutr.

[5] Piccolomini A, Iskandar M, Lands L, Kubow S (2012). High hydrostatic pressure pre-treatment of whey proteins enhances whey protein hydrolysate inhibition of oxidative stress and IL-8 secretion in intestinal epithelial cells. Food Nutr Res.

[6] Chatterton DEW, Smithers G, Roupas P, Brodkorb A (2006). Bioactivity of β lactoglobulin and α-lactalbumin–technological implications for processing. Int Dairy J.

[7] Kokuba H, Aurelian L, Neurath AR (1998). 3-Hydroxyphthaloyl-β-lactoglobulin IV antiviral activity in the mouse model of genital herpesvirus infection. Antivir Chem Chemother.

[8] Takakura N, Wakabayashi H, Ishibashi H, Teraguchi S, Tamura Y, Yamaguchi H (2003). Oral lactoferrin treatment of experimental oral candidiasis in mice. Antimicrob Agents Chemother.

[9] Verma AR, Vijayakumar M, Mathela CS, Rao CV (2009). In vitro and in vivo antioxidant properties of different fractions of Moringa oleifera leaves. Food Chem Toxicol.

[10] Sreelatha S, Padma PR (2009). Antioxidant activity and total phenolic content of Moringa oleifera leaves in two stages of maturity. Plant Foods Hum Nutr.

[11] Coppin JP, Xu Y, Chen H, Min Hsiung P, Chi Tang H, Rodolfo J (2013). Determination of flavonoids by LC/MS and anti-inflammatory activity in Moringa oleifera. J Funct Foods.

[12] Tong L, Nelson N, Tsourigiannis J, Mulligan AM (2011). The effect of prolonged fixation on the immunohistochemical evaluation of estrogen receptor, progesterone receptor, and HER2 expression in invasive breast cancer: a prospective study. Am J Surg Pathol.

[13] Giustina A, Mazziotti G, Canalis E (2008). Growth hormone, insulin-like growth factors, and the skeleton. Endocr Rev.

[14] Jawad MH, Al-Hijazi, AY (2015). Histological and mechanical evaluation of the osseointegration of titanium implants by the modifications of thread design and/or coating with flaxseed (An experimental study on rabbits). J Bagh Coll Dent.

[15] Sugihartini N, Fajri A, Rahmawati DR (2018). Formulation of Moringa oleifera leaf extract in lotion and gel as sunscreen.

[16] Al Molla BH, Al Ghaban N, Taher A (2014). Immuno-histochemical evaluation: The effects of propolis on osseointegration of dental implants in rabbit's tibia. J Dent Res Rev.

[17] Mahmood MS, Al Ameer SS (2017). Assessment of calcium carbonate coating on osseointegration of commercially pure titanium implant by torque removal test and histomorphometric analysis. J Bagh Coll Dent.

[18] Mohamed IF, Ghani BA, Fatalla AA (2022). Histological evaluation of the effect of local application of punica granatum seed oil on bone healing. Int J Biomater..

[19] Kamil NB, Majeed SS, Salman MA (2022). Histological effect of artichoke leaf extract on bone healing in rats. J Contemp Med Sci.

[20] Mohamed MAH (2013). The effect of autologous bone marrow-derived stem cells with estimation of molecular events on tooth socket healing in diabetic rabbits (a histomorphometric, histological and immunohistochemical experimental study. J Bagh Coll Dent.

[21] Pandey C, Rokaya D, Bhattarai BP (2022). Contemporary concepts in osseointegration of dental implants: a review. Biomed Res Int..

[22] Timothy E L (2017). Application of whey protein isolate in bone regeneration: Effects on growth and osteogenic differentiation of bone-forming cells. J Dairy Sci.

[23] Rodríguez Carballo E, Gámez B, Ventura F (2016). P38 MAPK signaling in osteoblast differentiation. Front Cell Dev Biol..

[24] Zhang DW, Cheng Y, Wang NL, Zhang JC, Yang MS, Yao XS (2008). Effects of total flavonoids and flavonol glycosides from Epimedium koreanum nakai on the proliferation and differentiation of primary osteoblasts. Phytomedicine.

[25] Othman Jassim H, Al-Ghaban NMH (2023). Effect of eucommia ulmoides on healing of bon defect using histological and histomorphometric analysis in rat: in vivo study. Arch Razi Inst.

[26] Davison NL, ten Harkel B, Schoenmaker T, Luo X, Yuan H, Everts V (2014). Osteoclast resorption of beta-tricalcium phosphate controlled by surface architecture. Biomaterials.

[27] Corrochano AR, Buckin V, Kelly PM, Giblin L (2018). Invited review: Whey proteins as antioxidants and promoters of cellular antioxidant pathways. J Dairy Sci.

[28] Verma AR, Vijayakumar M, Mathela CS, Rao CV (2009). In vitro and in vivo antioxidant properties of different fractions of Moringa oleifera leaves. Food Chem Toxicol.

[29] Mohammad MH, Al-Ghaban NMH (2018). Histological and histomorphometric studies of the effects of hyaluronic acid on osseointegration of titanium implant in rabbits. J Bagh College Dentistry.

[30] Rusu D, Drouin R, Pouliot Y, Gauthier S, Poubelle PE (2010). A bovine whey protein extract stimulates human neutrophils to generate bioactive IL-1Ra through a NF-κB and MAPK dependent mechanism. J Nutr.

[31] Hassan MAA, Al-Ghaban NMH (2020). Immunohistochemical localization of bone morphogenic protein-2 in extracted tooth socket treated by local application of grape seeds oil in rabbits. Biochem Cell Arch.

[32] Minj S, Anand S (2020). Whey proteins and their derivatives: bioactivity, functionality, and current applications. Dairy.

[33] Ibrahim MEED, Alqurashi RM, Alfaraj FY (2022). Antioxidant activity of Moringa oleifera and Olive Olea europaea L leaf powders and extracts on quality and oxidation stability of chicken burgers Antioxidants.

[34] Mohamed IF, Ghani BA, Fatalla AA (2022). Histological evaluation of the effect of local application of Punica granatum seed oil on bone healing. Int J Biomater..

[35] Lean JM, Mackay AG, Chow JW, Chambers TJ (1996). Osteocytic expression of mRNA for c-fos and IGF-I: an immediate early gene response to an osteogenic stimulus. Am J Physiol.

[36] Sheng MHC, Zhou XD, Bonewald LF, Baylink DJ, Lau KHW (2013). Disruption of the insulin-like growth facotor-1 gene in osteocytes impairs developmental bone growth in mice. Bone.

[37] Saini V, Marengi DA, Barry KJ, Fulzele KS, Heiden E, Liu X (2013). Parathyroid hormone (PTH)/PTH-related peptide type 1 receptor (PPR) signaling in osteocytes regulates anabolic and catabolic skeletal responses to PTH. J Biol Chem..

[38] Fatrai S, Elghazi L, Balcazar N, Cras-Meneur C, Krits I, Kiyokawa H (2006). Akt induces β-Cell proliferation by regulating cyclin D1, cyclin D2, and p21 levels and cyclin-dependent kinase-4 activity. Diabetes.

[39] Duan J, Yang Y, Zhang E, Wang H (2020). Co-Cr-Mo-Cu alloys for clinical implants with osteogenic effect by increasing bone induction, formation and development in a rabbit model. Burns Trauma..

[40] Saad KAE, Abu-Shahba AGT, El-Drieny EA, Khedr MS (2015). Evaluation of the role of autogenous bone-marrow–derived mesenchymal stem cell transplantation for the repair of mandibular bone defects in rabbits. J Craniomaxillofac Surg.

[41] Shayegan A, Petein M, Abbeele AV (2008). Beta-tricalcium phosphate, white mineral trioxide aggregate, white Portland cement, ferric sulfate, and formocresol used as pulpotomy agents in primary pig teeth. Oral Surg Oral Med Oral Pathol Oral Radiol Endod.

